# Not “just necessity”? Two-x-eco-cultural dilemmas and the ethnobiological importance of the informal grannies’ markets in Moldova

**DOI:** 10.1186/s13002-025-00770-8

**Published:** 2025-03-11

**Authors:** Andrea Pieroni, Dauro Mattia Zocchi, Mousaab Alrhmoun, Naji Sulaiman, Miroslava Bavorova, Renata Sõukand

**Affiliations:** 1 University of Gastronomic Sciences, Piazza Vittorio Emanuele II 9, 12042 Pollenzo, Italy; 2 Department of Medical Analysis, Tishk International University, Erbil, Kurdistan 44001 Iraq; 3 Dipartimento Di Lingue, Letterature E Culture Straniere, University of Bergamo, Piazza Rosate 2, 24129 Bergamo, Italy; 4 Faculty of Agricultural, Environmental and Food Sciences, Free University of Bolzano, Piazza Università 5, 39100 Bolzano, Italy; 5 Faculty of Tropical AgriSciences, Czech University of Life Sciences Prague (CZU), Kamýcká 129, 16500 Praha, Suchdol, Czech Republic; 6 Institute of Agricultural Economics and Information (UZEI IAEI), Mánesova 75, 120 58 Prague, Czech Republic; 7 Department of Environmental Sciences, Informatics, and Statistics, Ca’ Foscari University of Venice, Via Torino 155, 30174 Venice, Italy

**Keywords:** Ethnobiology, Food heritage, Informal markets, Grannies’ markets, Moldova

## Abstract

Informal food markets, particularly those managed by (elderly) women in post-communist Eastern Europe, represent a biocultural phenomenon of profound significance since globalisation and increasingly strict legal frameworks often threaten these reservoirs of biocultural food heritage. In the fall of 2022 and 2023, a preliminary field study was conducted by visiting the informal markets of six Moldovan centres: Chișinău, Orhei, Bălți, Călărași, Comrat, and Taraclia, and conversing with approximately 40 mid-aged and elderly sellers. We argue that these markets are crucial in sustaining small-scale farming, preserving biodiversity, and maintaining a connection between urban communities and rural communities and, ultimately, between these rural citizens and their nature, keeping small-scale family farming and domestic traditional gastronomic activities alive. By trading fresh, homegrown, and homemade food and goods (including handicrafts), these mid-aged and elderly vendors support local economies, promote environmental sustainability, and safeguard traditional ecological knowledge and cultural heritage. This paper explores how grannies’ markets contribute to biocultural diversity and sustainable food practices, especially amid the country’s recent turbulent political, socioeconomic, and demographic challenges. The analysis advocates for the survival rights of these ecological, economic, and cultural (2-x-eco-cultural) refugia and invites ethnobiologists, food studies and cultural heritage scholars, rural sociologists, and agricultural economists to defend the biocultural diversity of informal food markets, moving them from an “out of necessity” status to a solid pillar of a possible future, new, family farming and small-scale ecological and gastronomic (conscientious) tourism. Policymakers should protect and enhance these informal spaces, especially the socioecological farming systems behind them, as essential socioeconomic and environmental assets. They should emphasise their importance as hubs for biological diversity, cultural preservation, community cohesion, and ecological sustainability.

## Science highlights

- Informal food markets are bio-cultural spaces fostering ecological practices and community bonds
- Benefits of such markets and exchange extend far beyond the economic realm
- Markets continue to thrive nowadays, mainly due to the dedication of their elderly participants
- Supportive urban consumers perceive the food items sold there as healthier and more natural
- Markets exist due to economic struggles yet irradiate biocultural values, generating food security

## Policy and practice recommendations

- Ageing vendor populations require European, national, and regional policy interventions
- Local authorities must recognise and support the biocultural value of these markets
- Supporting and sustaining small-scale farming activities should be a priority for policymakers
- Educational programs raising awareness of these markets and their importance should be created
- Agritourism centred around these markets could offer sustainable 2-x-eco-cultural pathways

The importance of the biocultural heritage preserved by informal farmers’ food markets in a few Eastern European countries has been the focus of a work that we published before the COVID-19 pandemic [1]. Continuing this work, we analysed in this preliminary study Moldovan small informal markets, often managed by elderly women affectionately referred to as *bătrânele* (translated in English as grannies), a vital socioeconomic phenomenon in the country. These markets are more than simple places of trade; they serve as hubs of cultural preservation, economic survival, and community interaction. Despite their informal nature, these markets fulfil critical roles within Moldova’s economy and society. This analysis explores their importance for biocultural diversity, focusing on gastronomic biodiversity, cultural resilience, and fragility since they mainly live in a perennial “out of necessity” status. This issue is particularly relevant to the Republic of Moldova as it is experiencing a critical time in which the crossroad between the path of future accession to the EU and another “eastern” trajectory pushed by enormous external political pressure is a great dilemma that dramatically divides the population.

The Republic of Moldova has been facing three decades of remarkable economic difficulties, and older adults, especially elderly smallholder farmers in rural areas of the country, are the poorest age group in the EU. Additionally, the deficit in the social security budget has directly affected pensioners’ well-being, and soon, Moldova may face the risk of becoming a ghost country of orphan older adults, left behind without the support of younger family members who have migrated to urban areas or abroad looking for job opportunities and better life [2].

With pensions often inadequate to meet basic needs, informal markets have started to provide and are providing a lifeline for elderly women. As in other post-communist countries, many of these grannies have worked in agriculture or other low-paying jobs, leaving them minimal savings. Their pensions, which sometimes fall below subsistence levels, necessitate other ways to supplement their income. Selling goods in informal markets became a survival strategy and a safety net a few decades ago, most possibly in the last phase of the communist dictatorship, allowing elderly sellers to maintain some financial independence in suddenly arrived capitalism [3, 4]. The informal markets have a more extended history. They were created to compensate for the low price of the state purchase of the portion of their produce; peasants in the territories occupied by the Soviet Union were allowed to sell their surplus products on the free market already in 1950 and consolidated this custom especially in the last years of the communist period.

**Fig. 1:**
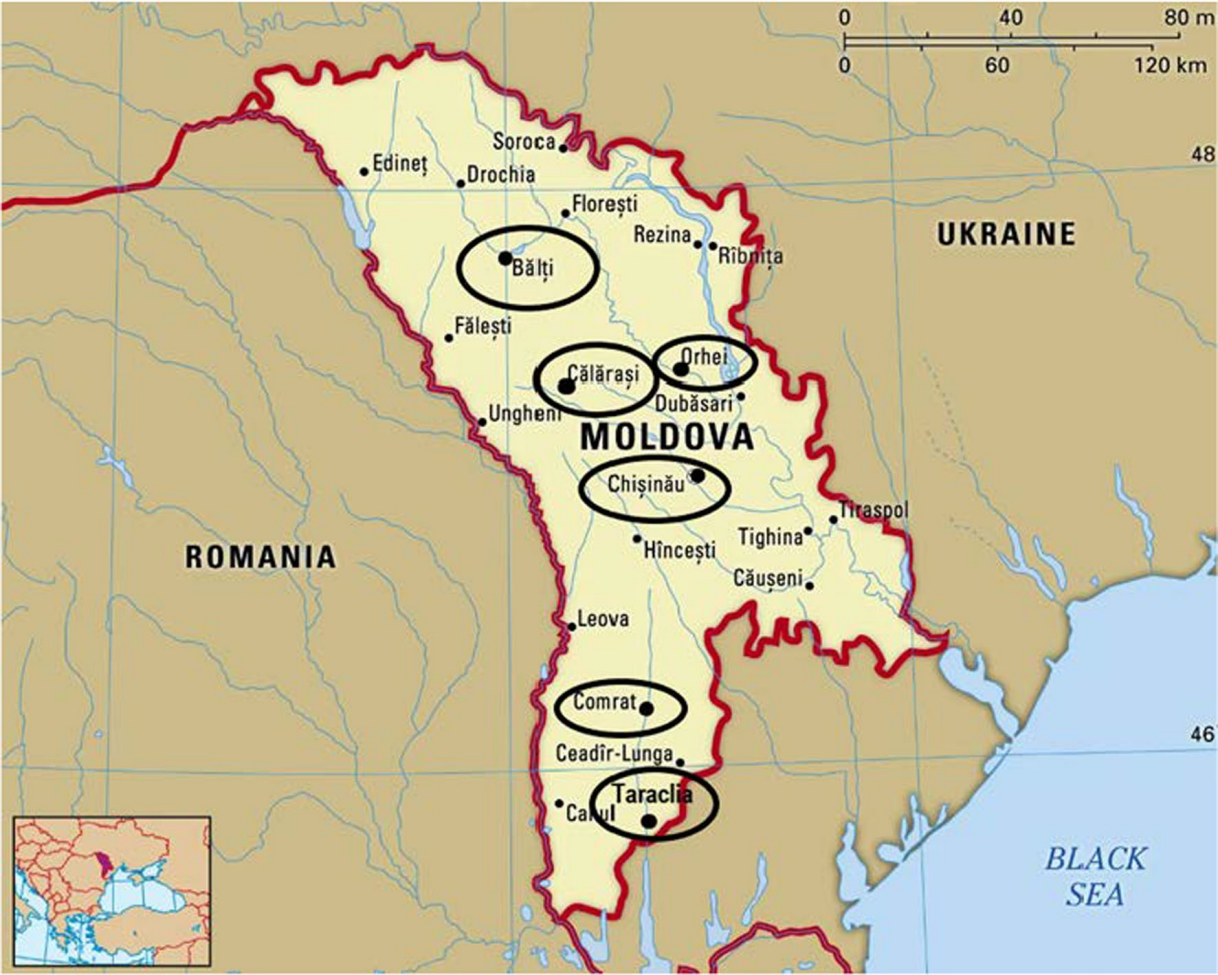
The visited centres in Moldova

This preliminary study was conducted by visiting the informal markets of six Moldovan centres: Chișinău, Orhei, Bălți, Călărași, Comrat, and Taraclia, via informal conversation with 40 old grannies (﻿Fig. 1). As usual in post-soviet countries, grannies’ informal markets are always located near the official farmers’ market, along the road and sometimes close to train and bus stations.

Despite a developing farm succession crisis [5], Moldova’s agricultural sector remains central to its economy (Moldova is by far the country in Europe with the highest number of people employed in the farming sector, approximately 54%; data of the Word Bank in 2022), and informal markets are a direct outlet for surplus produce from family farming that might otherwise go to waste. This local trade generates income for the old and mid-aged (often female) sellers and supports urban consumers seeking affordable, fresh, and organic food options.

**Fig. 2:**
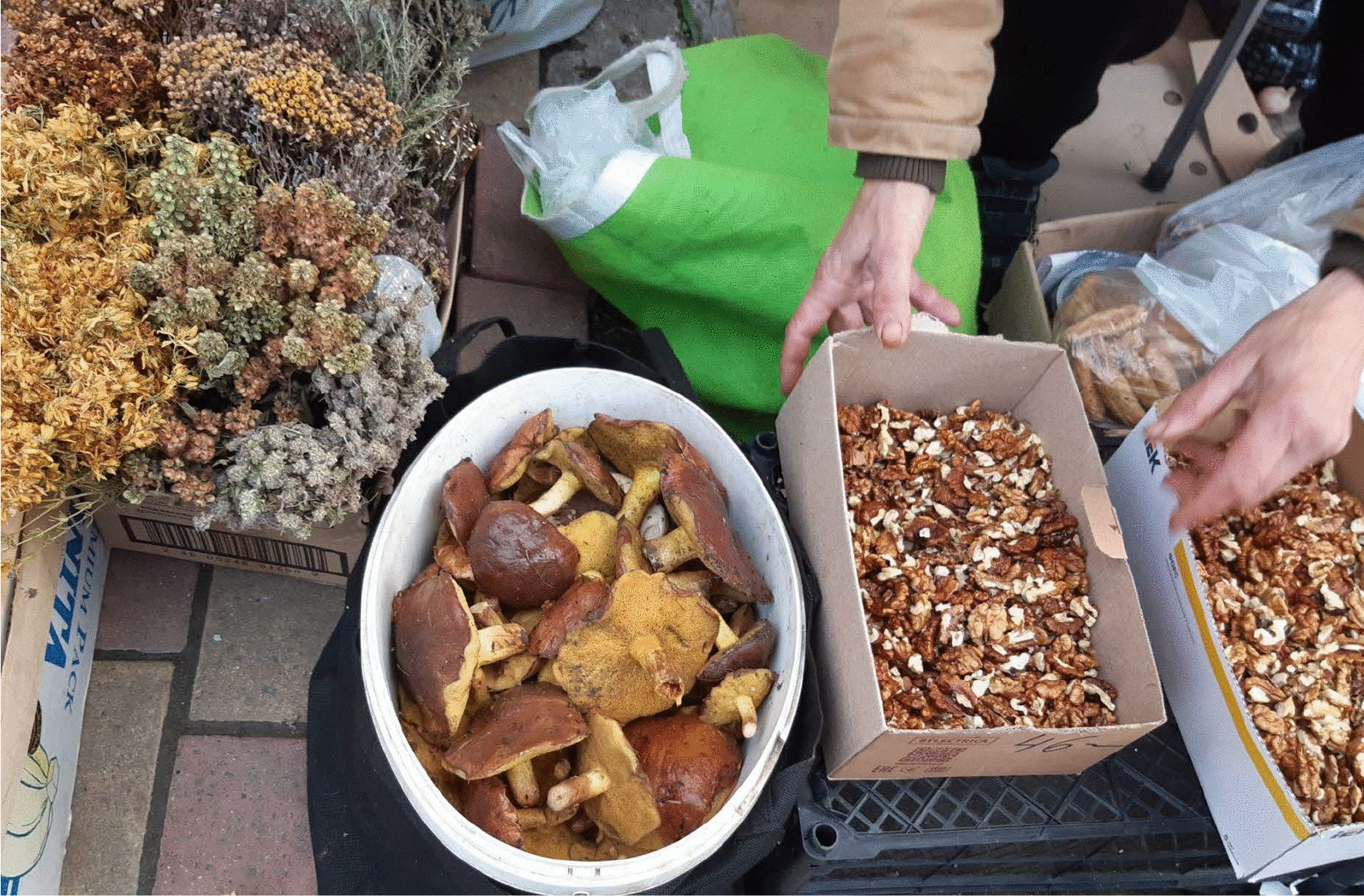
Dried various herbal teas, fresh *Suillus* mushrooms, and walnuts sold at the grannies’ informal market in Chișinău city centre

**Fig. 3:**
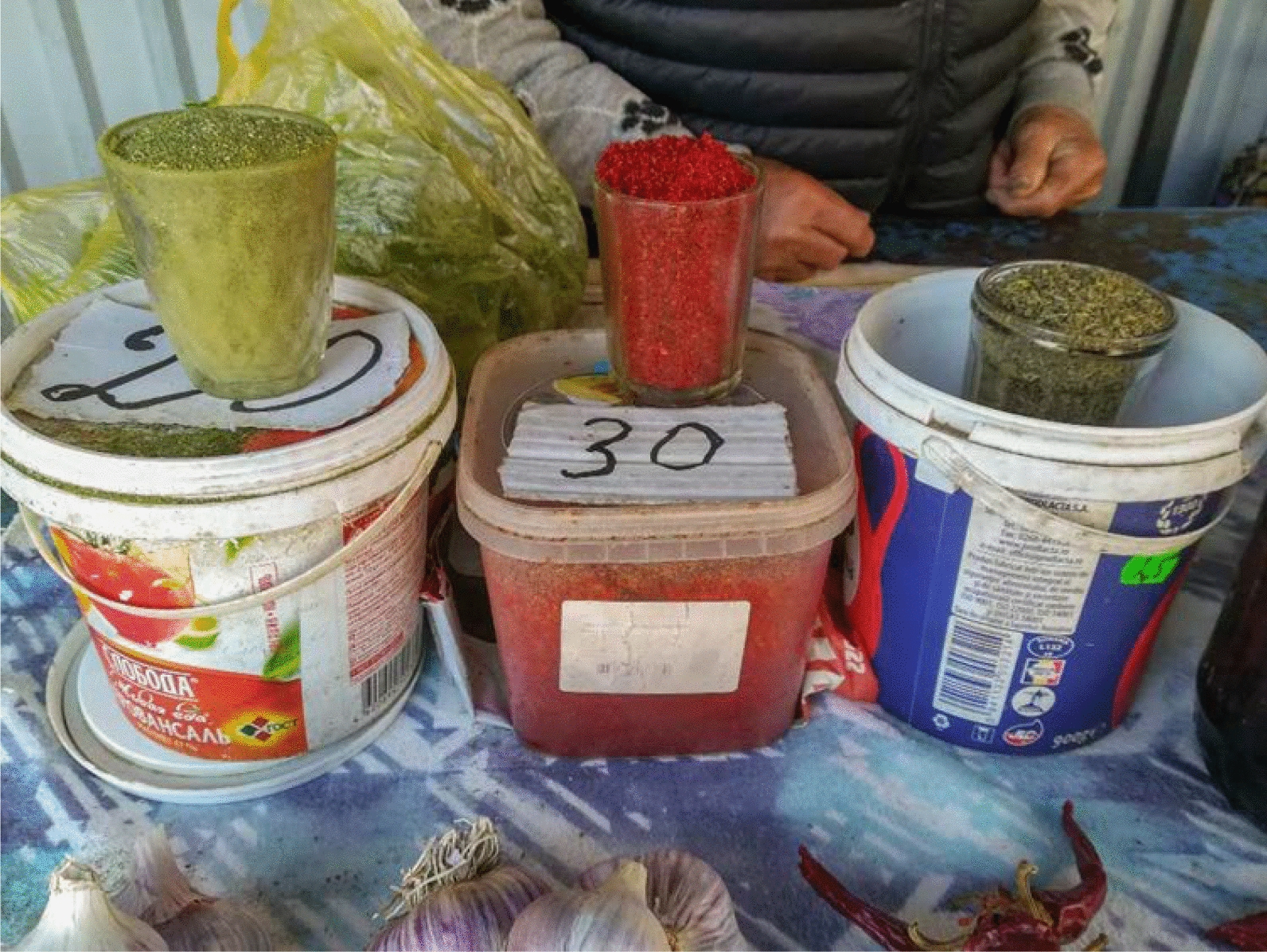
The tiny informal food market in Taraclia sells the *myrudia* mix and powdered red sweet paprika, two food heritage markers of the Bessarabian Bulgarian minority

**Fig. 4:**
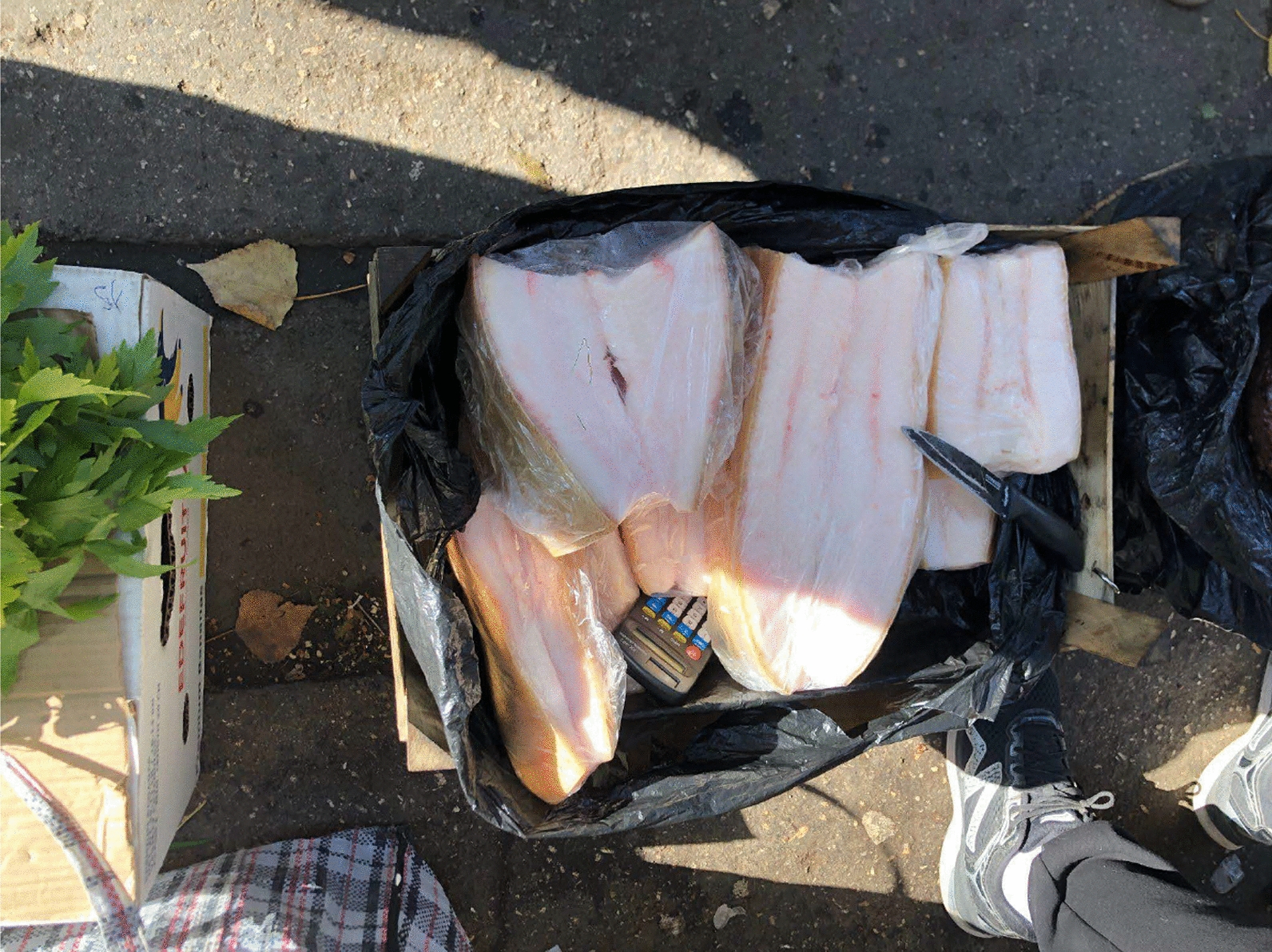
Homemade salted pork lard sold at the grannies’ informal market in Chișinău city centre, close to the central train station

**Fig. 5:**
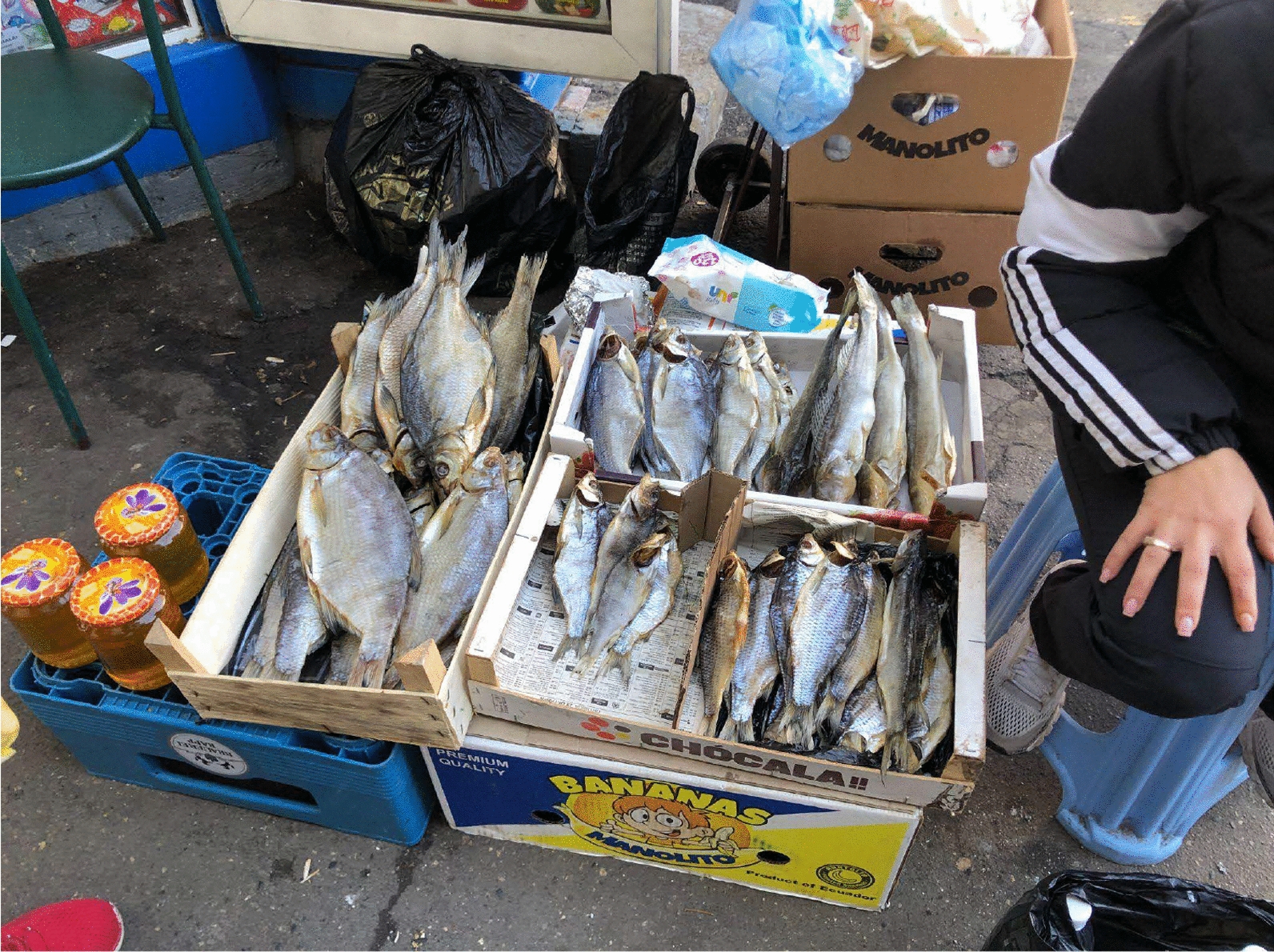
Dried freshwater fish sold at the grannies’ informal market in Chișinău city centre, close to the central train station

**Fig. 6:**
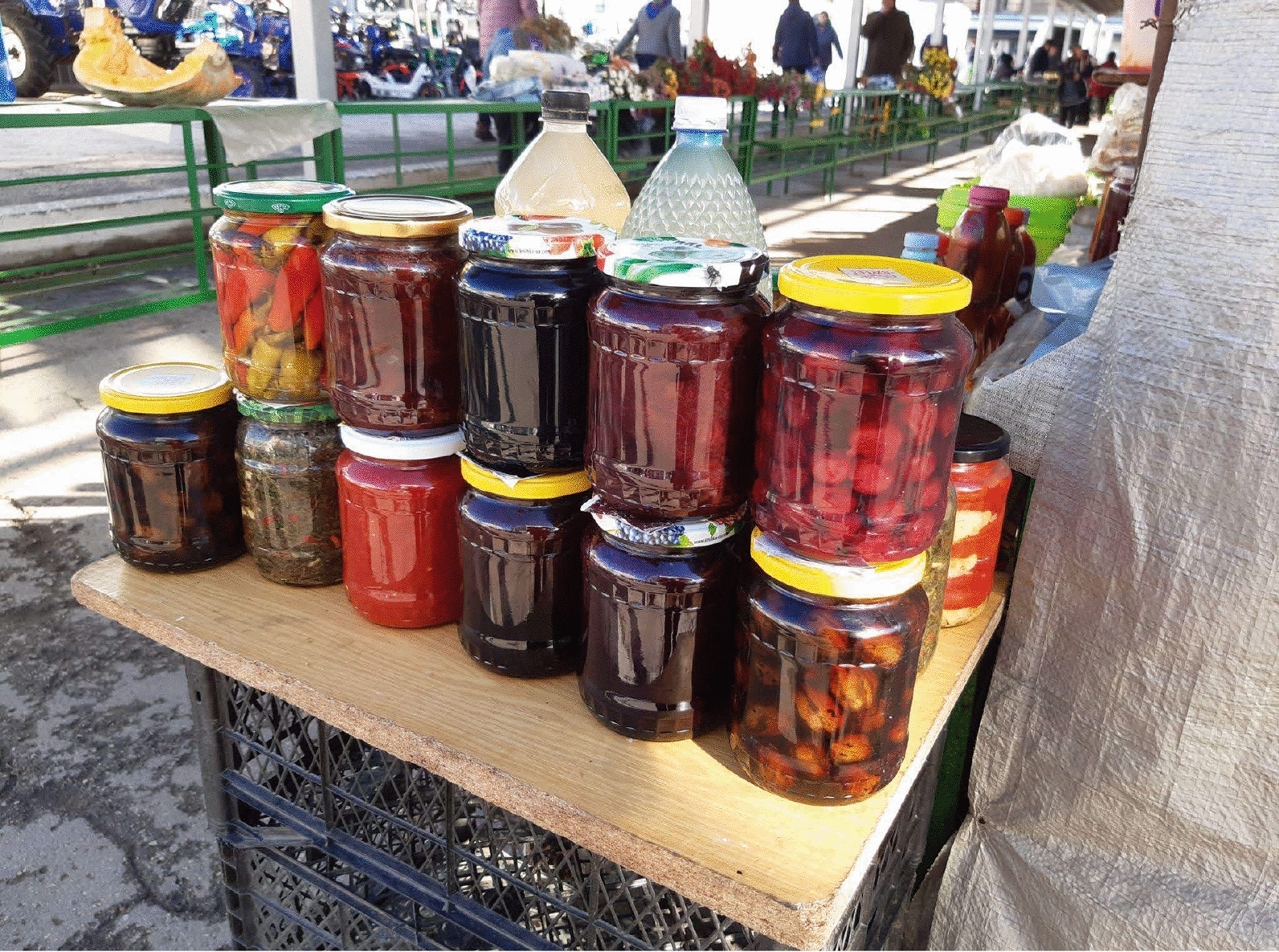
Various fruit and vegetable preserves sold in the semi-formal part of Chișinău city centre market

Moldova has Europe’s lowest per capita income in 2024 (together with Ukraine and Azerbaijan, IMF, 2024), and many residents rely on informal markets for budget-friendly goods. The grannies’ markets offer essential products at lower prices than formal retail outlets, partly because of lower overhead costs and direct sales from producers. For many Moldovan families, especially those in rural or economically disadvantaged areas, these markets are crucial for accessing fresh food and household items within their limited budgets [6] (Figs. 2, 3, 4, 5, and 6).

The food products sold typically include homemade or homegrown products such as fruits, vegetables, eggs, bread, pickles, dairy products, sweet preserves, wine, and cured and fresh meat (see Table 1). The items shown in Table 1 demonstrate a wide variety of food ethnobiological treasures, most possibly preserved in *biocultural refugia* [7, 8].

**Table 1:** A few commonly sold food and herbal ingredients and products are recorded in the grannies’ informal markets of the six considered Moldovan centres. X: occurrence, XX: widespread occurrence

Name of the food/herbal ingredient or product	Surplus/Artisanal production/Collected from the Wild/Cultivated	Chișinău	Orhei	Bălți	Călărași	Comrat	Taraclia
*Adjika*	A/S	xx	x				
Bilberry jam	S	x	x	x			
Blackthorn (*Prunus spinosa* L.)	W/C	xx	xx	xx			
Blood sausages	A	xx	x	x			
Butter	A/S	xx	xx	xx	x	xx	x
Carp	S	x					
Clarified butter	A/S	x					
Cow milk	S/A	xx	xx	xx	x	x	
Curdle made from fresh milk	S/A	xx	xx	xx	xx	x	
Cured and smoked veal, beef	A	xx	xx	xx		x	
Dill preserved in salt	S	xx	x				
Diverse cuts of pork meat	S	x	x	x	x	x	x
Diverse dried herbaceous plants for teas (*Hypericum perforatum* L.*, Mentha* spp., *Equisetum arvense* L., etc.)	A	x		x	x	x	
Diverse fresh and dried mushrooms (esp. *Boletus* and *Suillus* spp.)	W	x	x	x		x	
Dried and smoked plums	A/S	xx	xx	xx	x	x	x
Dried cheese (brânză)	S/A	xx	xx	xx	x	x	
Dried cherries	A	x					
Dried mushrooms	S/A	x					
Dried salted fish	A	x			x	x	
Dried wild pears and apples	A	x	x		x	x	
Dry bread yeast	S/A	x				x	x
Fat of goat	S	x	x				
Fat of goose	S	x	x				
Fresh brined cheese (brânză)	S/A	xx	xx	xx	x	x	
Fresh/sour cream	S/A	xx	xx	xx	x	x	
Goat meat	S	x					
Goat milk	S/A	x					
Guelder rose (*Viburnum opulus* L.) fruits	W	x	x	x			
Guelder rose jam, compote	S	x			x	x	
Homemade pork conserves	S	x	x	x			
Homemade salted pork	S	x	x	x			
Horseradish	W	xx	xx	xx	x	x	x
Lactofermented watermelons	A	x			xx		
Meat jelly	A	x	x	x		x	
Mixed fruit compotes	S	x	x	x	x	x	
*Myrudia* (mixture of powdered dried herbs: *Trigonella caerulea* (L.) Ser. and *T. foenum-graecum* L., dill, mint, parsley, lovage, or other dried aromatic sppecies)	S/A/C	x					x
Osage orange (*Maclura pomifera* (Raf.) C.K.Schneid.)	W			x			
Paprika (powdered dried local belly peppers variety)	S/A/C	x					x
Parsley roots	C	xx	x	x			
Pickled cabbage	S/A	xx	x	x	x	x	
Pickled cucumbers	S/A	xx	xx	xx	x	x	
Pickled paprika	A	xx	x				
Pickled tomatoes (red and green)	S/A	xx	xx	xx	x	x	
Pickled/salted grape leaves	S	xx	x	x	x		
Preserved horseradish	A/S	xx	x	x			
Raspberry jam	S	x	x	x	x		
Red and white *Atriplex hortensis* L. landraces	C/W		xx				
Rose petals jam	S	x	x				
Salted pork	A	xx	x	x			
Salted/marinated mushrooms	S	xx	xx	x	x	x	
Sausages	A	x	x	x			
Sheep or lamb meat-based preserves	A					x	
Smoked fish	A	x					
Smoked pork	A	xx	xx	xx			
Sour milk	S/A	xx	xx	xx	xx	x	
Thornbush and hawthorn fruits (*Rubus* and Crataegus spp.)	S	x			x		
Variety of juices	S	x	x		x		
Veal maw (cheese starter)	A	x	x				
Walnuts	S	xx	xx	xx	x	x	x
Wine (homemade)	S	x	x	x			x

The food ingredients and products sold in the grannies’ market come directly from the sellers’ small garden plots or traditional skills passed down through generations.

The grannies leverage these resources to turn their labour and knowledge into tangible economic benefits. Most importantly, these markets are a very intense interface between rural and urban citizens, and urban consumers’ expectations and requests may also shape and stimulate local food productions or preparations of specific items, creating further opportunities for small-scale producers and farmers, as already observed elsewhere [9, 10].

**Fig. 7:**
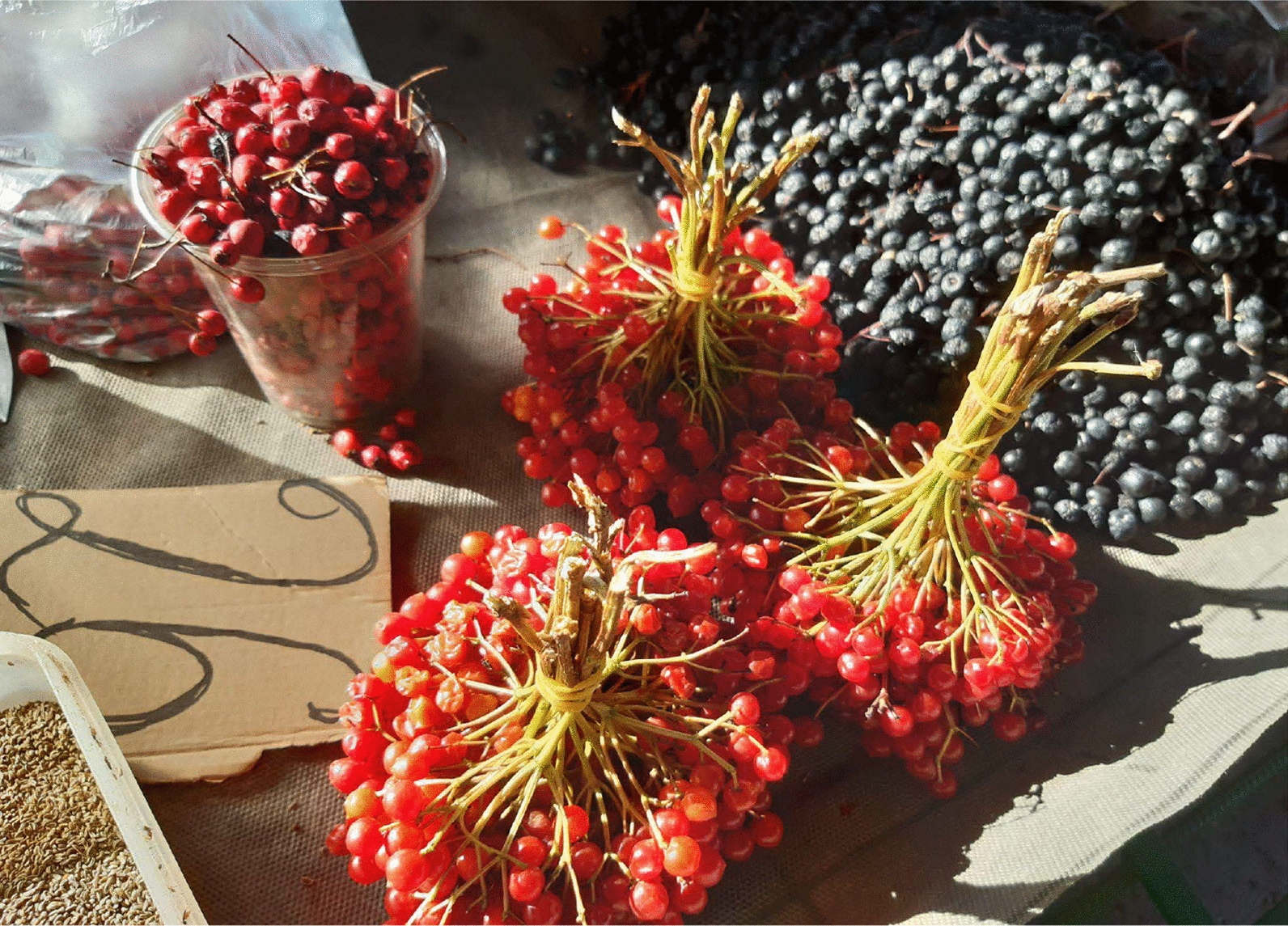
Guelder rose berries, hawthorn, and aronia fruits sold in Chișinău city centre market

**Fig. 8:**
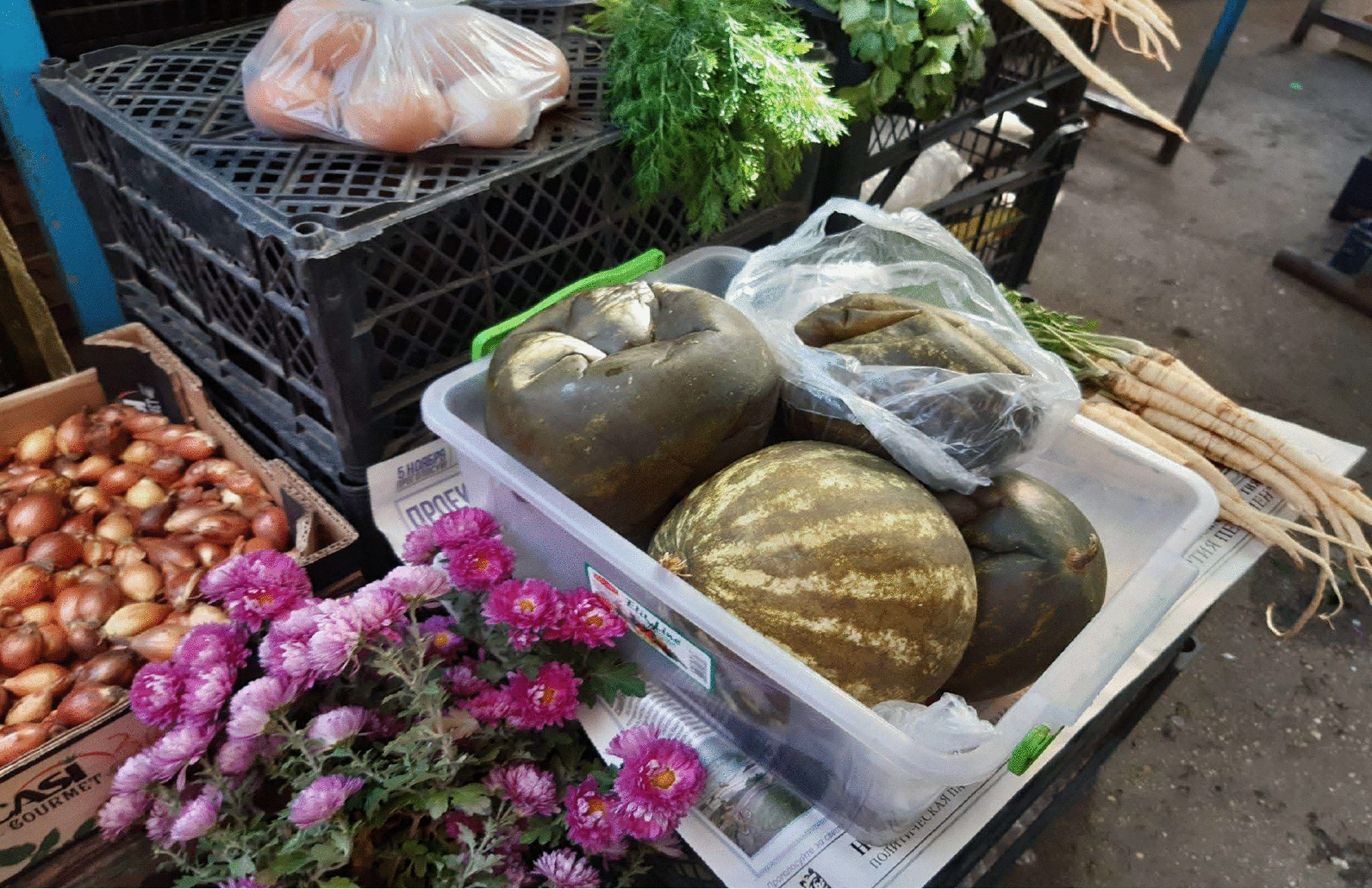
Lactofermented watermelons sold among home-produced onions, ornamental flowers, eggs, parsley roots, and dill in Bălți central market

**Fig. 9:**
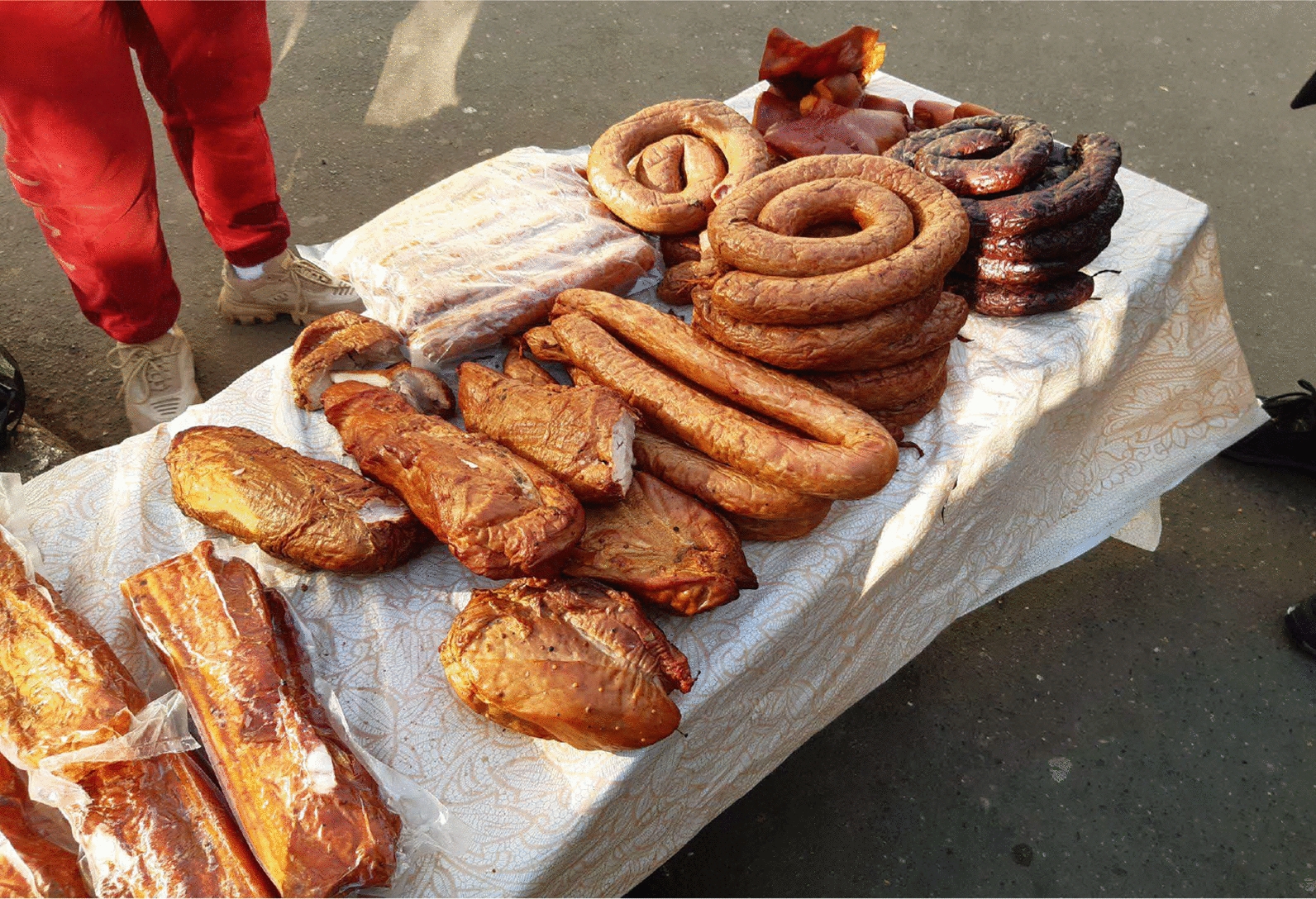
Various artisan meat products are sold in the informal part of the Chișinău market

Table 1 shows the goods sold in these markets reflect Moldova’s rich cultural heritage. Grannies preserve traditional culinary practices by selling homemade vegetables, fruits (both cultivated and wild), wild greens, mushrooms, fresh and cured homeproduced meat, dairy products, pickles, and preserved and baked goods (Figs. 7, 8, and 9).

We list hereby a few distinctive elements of the Moldovan food heritage that we could observe in the grannies’ markets: elongated red beet landraces, parsley roots, dill, lovage, wild sorrel, and dock; various landraces of apples, quince, plums, cherry, and sour cherries; homemade porc (smoked) sausages; fresh duck and goose meat, goat meat, fresh carps; and various *brânză* cheese.

The food heritage of the Bulgarian and Gagauz minorities, primarily found in the markets of Taraclia and Comrat, is fascinating: Bessarabian Bulgarian traditional food ingredients and products at the market include red paprika powder prepared by sun-drying local landraces of belly peppers, *myrudia* (a mix of powdered aromatic dried herbs generally used on freshly baked bread and other dishes and based mainly on *Trigonella caerulea* (L.) Ser. and *T. foenum-graecum* L. aerial parts), the archaic dried wild hop-based and wine-based bread yeast (made with wild hop inflorescences and wine tartrate) [11], and the ceremonial, very fluffy, Bulgarian wild-hopped bread (prepared with the yeast as mentioned earlier); Gagauz markets offer instead a distinct homemade lamb or cow meat-based aspic called *kavurma*.

Additionally, grannies sometimes sell non-food items at the same place, such as handicrafts like embroidered textiles and knitted items. Through their work, these elderly women ensure that cultural knowledge is not lost. They serve as informal custodians of Moldova’s heritage, passing on traditions to younger generations and showcasing them to the broader community. Moreover, Moldova’s rural identity is deeply intertwined with agriculture, and these markets exemplify this connection. Many grannies selling in these markets grew up in farming communities and relied on small-scale traditional horticulture and, more rarely, shepherding. Their adherence to non-industrialised, large-scale farming practices, such as traditional cultivation methods and seed preservation, aligns with growing global trends towards sustainable agriculture. In this way, the markets preserve cultural identity and contribute to environmental sustainability [12].

Beyond their economic function, these markets serve as important social spaces. They provide an opportunity and a socialisation space for both the sellers and the urban consumers/producers, many of whom live alone or experience social isolation, to engage with the community. These interactions foster a sense of purpose and belonging, counteracting the loneliness often accompanying ageing. For customers, these markets are more than transactional spaces where trust and relationships are built. Regular customers frequently rely on consolidated personal relationships of trust with the sellers, creating a warm and familiar atmosphere absent in modern supermarkets and shops. According to our observations, these markets also facilitate intergenerational exchanges. Younger consumers and sellers interact with older generations, learning from their experiences and gaining insights into traditional practices. This interaction bridges generational gaps, fostering mutual respect and understanding within the community [13].

One of the defining features of informal markets is their need for more official regulation. While this flexibility allows them to thrive, it also exposes participants to risks. Grannies operating in these markets often lack legal protection, making them vulnerable to harassment or forced eviction by authorities, which, however, are generally very tolerant, as we can see in the field. Additionally, they do not have access to social security benefits or health insurance linked to their work, leaving them unprotected in case of illness or economic downturns. The ageing demographic of sellers poses a long-term challenge. Despite declining health, many grannies endure physically demanding work transporting goods, standing for hours, and enduring harsh weather conditions. As Moldova’s population continues to age and few young people want to engage in food and agricultural activities [14], the sustainability of these markets depends on finding ways to support elderly sellers.

With expanding supermarkets and formal retail outlets, informal markets face growing competition. Younger consumers often prefer the convenience of modern shopping options, which can lead to declining foot traffic in traditional markets [15, 16]. However, the personal touch and authenticity offered by the grannies remain a unique selling point that large retailers need help to replicate.

Governments and local authorities should be urged to provide the eventual recognition of informal markets’ biocultural and food heritage-centred contribution to the economy and society by offering supportive policies to sustain the continuity of practice. We offer a few practical steps to address:

- Creating designated spaces for informal markets would provide grannies with secure and stable operating locations.
- Waiving or reducing licensing fees for elderly traders could alleviate financial pressures.
- Improving access to healthcare and social security for elderly women working in informal markets is critical. Policymakers could design programmes specifically aimed at this demographic, such as subsidised health services or pension top-ups for those engaged in informal trading.
- Promoting these markets as cultural and tourist attractions could increase their visibility and attract a wider audience. Efforts highlighting the uniqueness of the goods, such as organic produce and traditional crafts, would enhance their appeal to locals, visitors, and young Moldovan generations.
- Basic training in marketing, financial literacy, food safety, and product packaging could help these traders increase their competitiveness.
- Workshops on digital tools and online marketplaces might also open new avenues for selling their products, particularly for younger family members who may assist in the trade.

The persistence of informal food markets, particularly those run by grannies, represents economic resilience and a profound connection to Moldova’s biocultural identity. These markets serve as cultural reservoirs where traditional agricultural practices and culinary heritage are maintained, ensuring continuity amid rapid societal and economic changes. Linking their role to broader academic frameworks addressing informal economies, biocultural diversity, and sustainability is essential to fully appreciating their value.

Informal markets exemplify the concept of “biocultural refugia”, as Barthel et al. [7] theoretically articulated, and Pieroni and Sõukand [8] outlined in an Azeri case study, where local ecological knowledge and traditional food practices are preserved. By cultivating heirloom crops, sustainably harvesting wild food plants, and producing homemade artisanal food products (cured meat, dairies, preserves, pickles, and bread), grannies contribute to global food sovereignty efforts [17], reinforcing local control over food systems aligned with sustainable development goals [18]. These markets also embody the principles of short-food chains, emphasising the importance of local food systems prioritising ecological and cultural values over industrial efficiency. As argued by Renting et al. [19], the short supply of informal markets reduces the environmental costs associated with long-distance transportation and excessive packaging. This aligns with the growing movement towards sustainable consumption and production patterns advocated by the United Nations’ Agenda 2030.

However, this market seems often to emerge out of their “status of necessity”, and it is unclear if their future could be independent of this necessity. The Journal of Ethnobiology and Ethnomedicine recently addressed one of its debates starting from the reflection of Hartel et al. [20], in which authors postulated that Traditional Ecological knowledge (TEK) is often maintained not because of positive values about the environment but because of poverty and a lack of options. Hanazaki [21] replied in her essay that this oversimplified causal argument might be based on a colonialist perspective of what a less advantageous circumstance is; she further sustained how this conundrum often ignores the struggles and resistance of traditional knowledge holders and the urgent call for socioenvironmental justice.

The Moldovan grannies market exemplifies this puzzle: on the one hand, the markets exist because of rural households’ economic struggles, and on the other hand, they irradiate biocultural values and generate food sustainability and sovereignty [22].

The knowledge transfer facilitated by these markets already ensures that urban generations are exposed to traditional farming and food preparation methods. However, the younger Moldovan generations are leaving the country unprecedentedly. This interaction between generations could be critical in countering the homogenising effects of globalisation, which threaten to erode local diversity of knowledge and with it, also the *dark*, hardly documentable part of it [23]. Hart’s work [24] on the informal economy underscores how such spaces foster trust and social cohesion, creating a sense of community that transcends mere economic transactions. The dilemma is, therefore, eminently 2-x-eco-cultural (i.e., ecological, economic, and cultural): Ecological and economical, since both terms relate to the sustainability/durability of the domestic food heritage, and cultural too, since the sense of food identity may play a role in shaping small-scale productions despite possible practical and economic adversities.

The ageing vendor population in Moldova presents a unique challenge that could require policy interventions at European, national, and regional levels. Governments and local authorities must recognise the biocultural value of these markets and implement supportive measures. Designated trading spaces, waived licensing fees, and support for elderly vendors could be crucial to ensuring their sustainability. Additionally, integrating these markets into the EU legal frames of food security could be challenging, even though cultural food and eco-tourism strategies (including small-scale speciality food and niche restaurants) could enhance their visibility and attract new audiences from abroad. However, these measures could be more problematic given the economic circumstances of the country and its governmental budget. Still, a future, possible access to the EU and its structural funds and financial sustain may paradoxically both enhance and mitigate these difficulties.

In conclusion, the informal food markets managed by grannies in Moldova are not merely economic entities but primarily vital biocultural spaces that sustain traditions, foster ecological practices, and strengthen community bonds. Their preservation is a matter of national significance and a global challenge, too, since hundreds of “Moldovan cases” can be found worldwide, reflecting the broader struggle of several rural and Indigenous communities to maintain their biocultural diversities and small-scale sustainable food systems within their economic struggles and an increasingly industrialised and homogenised world. As Gudeman [25] aptly noted, economies are not just systems of production and exchange; they are cultural expressions of how communities live, interact, and sustain themselves. These markets exemplify this ethos, making their support and preservation a pressing priority for ethnobiologists, policymakers, and local communities.

The informal markets managed by old grannies in Moldova have existed for the past three decades and are still essential to the nation’s sociocultural and economic fabric. They embody resilience, tradition, and community spirit, offering a lifeline to those involved while preserving Moldova’s food heritage. Despite their challenges, these markets continue to thrive nowadays, mainly due to the dedication of their elderly participants and the supportive and curious urban consumers, who perceive the food items sold there as much healthier and more natural than industrial supermarkets’ food.

Supporting and sustaining these markets, and especially the small-scale farming activities and the immense human-nature daily multiple interactions behind them, should be a priority for ethnobiologists, various food stakeholders (e.g., chefs, tourism managers, consumers, and foodies), and policymakers, as their benefits extend far beyond the economic realm, touching the heart of Moldovan rural communities’ sense of identity and community life. Initiatives such as educational programmes that raise awareness of these markets and their socioeconomic and cultural importance and promote agritourism centred around these markets could offer sustainable pathways. Nevertheless, the future of these markets is an interrogation mark, especially if we observe what happened with similar informal farmers’ markets in countries that entered the EU or are approaching it (most notably Romania and Albania), where these spaces were remarkably reduced or almost entirely disappeared. The dilemma of how grannies’ food markets can also be considered a witnessing, disappearing by-product of poverty until disadvantaged economic circumstances remain is at the core of the contemporary applied ethnobiological discourse; however, it may not be a mere, inevitable destiny.

## Data Availability

All data generated or analysed during this study are included in this published article.
